# A Molecular Diagnostic Tool to Replace Larval Culture in Conventional Faecal Egg Count Reduction Testing in Sheep

**DOI:** 10.1371/journal.pone.0037327

**Published:** 2012-05-22

**Authors:** Florian Roeber, John W. A. Larsen, Norman Anderson, Angus J. D. Campbell, Garry A. Anderson, Robin B. Gasser, Aaron R. Jex

**Affiliations:** 1 Faculty of Veterinary Science, The University of Melbourne, Parkville, Victoria, Australia; 2 Mackinnon Project, The University of Melbourne, Werribee, Victoria, Australia; The George Washington University Medical Center, United States of America

## Abstract

The accurate diagnosis of parasitic nematode infections in livestock (including sheep and goats) is central to their effective control and the detection of the anthelmintic resistance. Traditionally, the faecal egg count reduction test (FECRT), combined with the technique of larval culture (LC), has been used widely to assess drug-susceptibility/resistance in strongylid nematodes. However, this approach suffers from a lack of specificity, sensitivity and reliability, and is time-consuming and costly to conduct. Here, we critically assessed a specific PCR assay to support FECRT, in a well-controlled experiment on sheep with naturally acquired strongylid infections known to be resistant to benzimidazoles. We showed that the PCR results were in close agreement with those of total worm count (TWC), but not of LC. Importantly, albendazole resistance detected by PCR-coupled FECRT was unequivocally linked to *Teladorsagia circumcincta* and, to lesser extent, *Trichostrongylus colubriformis*, a result that was not achievable by LC. The key findings from this study demonstrate that our PCR-coupled FECRT approach has major merit for supporting anthelmintic resistance in nematode populations. The findings also show clearly that our PCR assay can be used as an alternative to LC, and is more time-efficient and less laborious, which has important practical implications for the effective management and control strongylid nematodes of sheep.

## Introduction

Strongylid nematodes of ruminants are responsible for substantial economic losses due to the diseases that they cause and the costs associated with their treatment and control [1]. These parasites impose a major financial burden on livestock industries globally. Small ruminants, such as sheep, can become infected with multiple strongylid nematodes, including species of *Teladorsagia*, *Trichostrongylus*, *Haemonchus*, *Nematodirus*, *Cooperia, Chabertia* and/or *Oesophagostomum* [2], which differ in their geographical distribution, pathogenicity and susceptibility to various anthelmintics [3].

The accurate diagnosis of nematode infections is central to their effective control, supports investigations into their epidemiology and ecology, and, importantly, can assist substantially in the monitoring of anthelmintic resistance in strongylid populations. Such resistance has emerged as a major economic and bionomic problem [4], predominantly as the result of an excessive and uncontrolled use of broad-spectrum anthelmintics (representing three main classes: benzimidazoles, imidazothiazoles and macrocyclic lactones). Although there has been a recent breakthrough in the development of a new drug, monepantel, representing an alternative compound class (amino-acetonitrile derivatives, AADs) [5], success in the discovery of new anthelmintics has been scarce over the last two decades [6]. Therefore, although there is hope for new, effective anthelmintics, there is also a major need to preserve compounds that we currently have at our disposal. Hence, monitoring the drug-susceptibility and -resistance status of strongylid nematode populations in livestock needs to be a high priority, and should underpin integrated management strategies.

Various *in vitro* methods, such as egg hatch- and larval development assays, have been used for estimating levels of drug-susceptibility/resistance in strongylid nematodes of small ruminants, cattle and horses. However, these assays can suffer from a lack of reliability, reproducibility and sensitivity [7]. The method most widely used to assess the efficacy of different anthelminthics in live sheep is the faecal egg count reduction test (FECRT) [8]. The diagnostic component of this test involves the enumeration of strongylid eggs in faecal samples before and after treatment of the animals with an anthelmintic compound. From the results, the percentage of reduction in the number of strongylid eggs per gram (EPG) following treatment provides an estimate of the susceptibility/resistance of nematode populations to a particular compound, and a population of worms is considered resistant if the reduction is <95% [9]. However, strongylid populations usually comprise multiple species, and it is, thus, not possible to assess the effect of a drug on different species in the populations, because eggs in faeces cannot be delineated to genus or species based on morphology (with the exception of *Nematodirus*). Therefore, the technique of larval culture (LC) is required to allow eggs to develop through to third-stage larvae (L3s), which can then be differentiated morphologically. However, LC has intrinsic limitations, which relate predominantly to the different requirements for hatching and larval development of individual nematode species [10], methodological differences among diagnostic laboratories, and the inability to unequivocally identify and differentiate particular genera and/or species [11].

There have been significant advances in establishing molecular methods for the genus- or species-specific diagnosis of strongylid infections in livestock [12]. Recently, we evaluated the performance of a PCR method for the diagnosis of naturally acquired strongylid nematode infections in sheep [13]. We established the diagnostic sensitivity (98%) and specificity (100%) of this assay by comparison with a conventional faecal flotation method, and also applied a system to rank the contribution of particular strongylid nematodes to EPGs in individual sheep with mixed-species infections. The ability to rapidly identify and rank nematodes according to their numerical contribution to observed faecal egg count results represents a major advantage over routine coprological methods, and shows clear potential to replace the conventional technique of LC. Therefore, we proposed that this PCR tool [13] can be used as a practical adjunct to conventional FECRT to enable the rapid inference of which species or genera of strongylid nematodes are susceptible or resistant to particular anthelmintic drugs. Here, we assess this tool for this purpose in a controlled experiment on sheep with naturally acquired infections of strongylids known to be resistant to benzimidazoles. We directly compared the results from the PCR evaluation with those obtained from routine LC and worm counts.

## Materials and Methods

### Experimental Design

The present study was conducted on a farm in Rokewood with owners permission [37°53′S/143°43′E], Victoria, Australia, with a known resistance problem in strongylid nematodes against one or more benzimidazoles; Relevant permission was granted from the owner of this farm to undertake this observational field study, which involved routine anthelmintic treatment of sheep and collection of faecal samples from sheep on this farm in Rokewood. This study was approved by the Animal Ethics Committee (AEC no. 0810850.1) of the University of Melbourne. Merino sheep (n = 80; 15 months of age; 36–59 kg; with ear tag identification) were available for FECRT and were shown previously to have average faecal egg counts of ≥150 EPG. Sheep were divided randomly into four groups (of 20 each), designated AB (albendazole-treated), ABC (albendazole-untreated control), MP (monepantel-treated) and MPC (monepantel-untreated control), respectively. For one sheep in group AB, no faecal sample was obtained after repeated sampling attempts, such that 19 samples could be collected. Groups AB and ABC were kept on the same pasture as were MP and MPC. Albendazole (Valbazen®, Coopers Animal Health) and monepantel (Zolvix®, Novartis) were administered orally by a qualified veterinarian using a syringe at a dose of 4.75 mg/kg (albendazole) and 2.5 mg/kg (monepantel), according to the bodyweight of the heaviest sheep in groups AB and MP, respectively. The experiment was conducted over a period of 13 days. Faecal samples were collected from sheep on days 0 and 10. Groups AB and MP were treated on day 0. A total number of 30 sheep (see subsection 2.3) were necropsied on day 13.

### Procurement of Faecal Samples and Conventional Coprological Testing

Fresh faecal samples (6.5–20 g) were collected directly from the rectum of individual sheep into plastic bags, chilled for transport and then stored at 4°C for a maximum of 1 week [14]. The numbers of small- to medium-sized (i.e. <100 µm in length and <50 µm in width), thin-walled ‘strongylid eggs’ per gram (EPG) of faeces were counted using a standard flotation method [15] with a theoretical detection limit of 10 EPG.

For each of the four experimental groups, an equal amount of faeces (2.5 g) from each individual sample was used to set up a 50 g composite LC in a plastic beaker. The cultures were incubated at 25°C for 10 days. L3s were then recovered by filling each beaker with water (25°C) and inverting it on to a Petridish [16]. The sheath extension lengths [17] of 100 L3s from each of the four cultures were measured to differentiate among *Teladorsagia*/*Trichostrongylus*, *Haemonchus* and *Chabertia*/*Oesophagostomum* L3s. Total lengths of L3s were measured to differentiate *Teladorsagia* from *Trichostrongylus*, according to the criteria of three different authors [18]–[20]. To further refine the delineation *Te. circumcincta* and *Trichostrongylus*, 50 L3s from each culture were exsheathed in aqueous hypochlorite (5%), and their caudal morphology examined for the presence/absence and number of tubercles [20].

### Total Worm Counts (TWC)

Three days following the second collection of faecal samples (on day 13), nine, nine, nine and three sheep were selected randomly from groups AB, ABC, MP and MPC, respectively, and then necropsied (approval granted through AEC no. 0810850.1). The entire gastrointestinal tract was removed from each of these sheep. Ligations were positioned anterior and posterior to the abomasum. TWC was performed as described by Anderson [21]. In brief, the entire contents of the abomasum and the proximal six meters of the small intestine were collected separately and each diluted in one litre of water. An aliquot (250 ml) thereof was fixed in formaldehyde (final concentration: 5%). The large intestine was opened longitudinally, distal to the spiral colon, and the worms recovered were fixed in 70% ethanol. Individual adult worms were identified morphologically to species according to Gibbons [22].

### PCR Testing

Genomic DNA from strongylid eggs, isolated from individual faecal samples, were column-purified and diluted (1/50) as described previously [23]. PCR-based testing was carried out as reported recently [13], employing primer pairs HAE-NC2, TEL-NC2, TRI-NC2, CHO-NC2 and OEV-NC2, in separate reactions, for the specific amplification from the second internal transcribed spacer (ITS-2) of nuclear ribosomal DNA from *Haemonchus contortus*, *Teladorsagia circumcincta*, *Trichostrongylus* spp., *Chabertia ovina* and *Oesophagostomum venulosum*, respectively. In addition, primer pair NC1–NC2 [24] was used, as a control, to assess inhibition in, and amplification efficiency for individual genomic DNA samples. Individual samples were identified as test-positive on the basis of the detection of an amplicon and also of a single, specific melt-peak that was consistent with that of an homologous control (for each PCR run). The specificity of the PCR, the cycling conditions and the products were verified by selective sequencing of amplicons using an established approach [25] and the subsequent comparison of individual sequence tags against known reference sequences for *Te. circumcincta*, *T. axei*, *T. colubriformis*, *T. vitrinus* and *C. ovina* (GenBank accession nos. AY439025.1, AY439026.1, AB503252.1, AY439027.1 and AY439021.1, respectively).

Any suspected inhibition in the PCR assay, potentially linked to faecal constituents (e.g., humic acids, phenolic compounds and/or polysaccharides), was assessed for all samples for which there was a discrepancy in results between faecal egg count and PCR. In brief, aliquots (2 µl) from samples that were test-negative by PCR, but were shown to contain strongylid eggs by coproscopic examination, were spiked with a limited amount (1 pg) of genomic DNA from *H. contortus* and then subjected to PCR. The amplification results from these aliquots were compared directly (in the same experiment) with that from 1 pg of *H. contortus* DNA alone and a sample without DNA (no-template control).

### Statistical Analysis

Samples were tested in conventional methods and PCR in a blinded manner. The reduction in EPG was calculated using the program RESO FECRT v4.0 (http://www.vetsci.usyd.edu.au/sheepwormcontrol/index.html). A population of stronglid nematodes was defined as resistant to an anthelmintic if the reduction in EPG was <95% and the lower confidence limit of the percentage of reduction was <90% [9]. The proportion of sheep that remained test-positive from day 0 to day 10 by PCR was compared between groups using Fisher’s exact test in the program Stata v.12.0 (StataCorp, USA). The performance (i.e., sensitivity, specificity and Kappa value) of individual PCR assays was calculated using an established approach [26]. The sensitivity and specificity as well as Kappa statistics of PCR were assessed in relation to results of TWC for 30 sheep, employing the program WinEpiscope 2.0 (http://www.clive.ed.ac.uk./winepiscope/); 95% confidence intervals (CI) for sensitivity and specificity values were calculated using the exact binomial method in Stata. The sensitivity of the specific PCRs for the detection of patent strongylid nematode infections was calculated by comparing the presence of adult female worms of individual species and the corresponding PCR results (because the PCR is based on the specific amplification of genomic DNA from thin-shelled strongylid eggs; [23]). The performance of the PCR assay using primer pair TRI-NC2 was calculated for all infected sheep as well as for those with a minimum TWC of ≥100 adult female *Trichostrongylus*.

## Results

### Results from FECRT Coupled to Conventional Coproscopic Testing and PCR

The coprological testing of 158 individual faecal samples collected from 79 sheep showed that 136 (86%) of these samples contained strongylid eggs. The arithmetic mean EPG in group AB decreased from 142 (day 0) to 41 (day 10), whereas there was no decrease in their corresponding (untreated) control group. The mean number of EPG in the group MP decreased from 177 (day 0) to 5 (day 10), whereas the numbers increased slightly in group-MPC (see Table 1) during the 10-day period. Based on this reduction in EPG numbers in groups AB and MP, calculated efficacies (with reference to their untreated control) were 64% (95% CI, 31–82%) and 97% (95% CI, 93–99%), respectively. For ten of the 136 samples with an EPG of 10–250, no PCR amplification was detected for any species. With the exception of one sample, these ten samples were from sheep that had received anthelmintic treatment (i.e., three, six and one sample from groups AB, MP and MPC, respectively) and all had an EPG of <50. Microscopic examination of the strongylid eggs in these samples indicated that they were damaged/degraded, with the exception of the sample from group MPC. Molecular screening by PCR showed that 135 (85%) faecal samples were test-positive for one or more of the target nematode species (which included *Te*. *circumcincta*, *Trichostrongylus* and *C. ovina*). Of these samples, there were nine, for which no strongylid eggs were detected by faecal flotation. The molecular analysis of 79 individual faecal samples collected on day 0 revealed that the largest percentage of test-positive faecal samples related to *Te. circumcincta* (84%), *Trichostrongylus* (92%) and, to a lesser extent, *C. ovina* (56%), which was a consistent pattern for all four groups on day 0 (Table 1). No sample was test-positive by PCR for *H. contortus* or *O. venulosum*.

**Table 1:** Results of coprodiagnostic testing.

		Day 0					Day 10				
	Groups^a^	ABC	AB	MPC	MP	Total (%)	ABC	AB	MPC	MP	Total (%)
Number of animals		20	19	20	20	79 (100)	20	19	20	20	79 (100)
Faecal egg count	positive	20	15	19	19	73 (92)	20	17	19	7	63 (80)
	Mean	117.5	142.1	182.5	177	–	115.5	41.1	199	5	–
	standard deviation	181.6	239.2	228.7	137.6	–	125.7	35.9	317.4	8.3	–
	Range	10–770	0–1070	0–760	0–530	–	20–530	0–120	0–1310	0–30	–
PCR positive		20	19	19	20	78 (99)	20	15	19	3	57 (72)
	*H. contortus*	0	0	0	0	0 (0)	0	0	0	0	0 (0)
	*T. circumcincta*	15	17	16	18	66 (84)	17	15	15	3	50 (63)
	*Trichostrongylus*	18	17	18	20	73 (92)	17	3	19	0	39 (49)
	*C. ovina*	10	8	12	15	45 (56)	13	0	15	0	28 (35)
	*O. venulosum*	0	0	0	0	0 (0)	0	0	0	0	0 (0)

Results from the testing of 158 individual faecal samples by conventional faecal egg count and species-specific PCRs using the primer pairs HC-NC2 (*Haemonchus contortus*), TEL-NC2 (*Teladorsagia circumcincta*), TRI-NC2 (*Trichostrongylus* spp.), CHO-NC2 (*Chabertia ovina*) and OEV-NC2 (*Oesophagostomum venulosum*). Shown are the number of egg count positive samples, mean eggs per gram, standard deviation, and range of strongylid egg counts recorded for the individual groups of sheep. Also shown are the number of species positive samples as determined by PCR for the different groups of sheep on days 0 and 10 of the experiment.

a Groups of sheep assigned as ABC (albendazole-untreated control), AB (albendazole-treated), MPC (monepantel-untreated control), MP (monepantel treated).

Using the PCR assay, 19 sheep in group AB were test-positive on day 0 and 15 sheep were test-positive on day 10. In contrast, all 20 sheep in group ABC were test-positive by PCR on days 0 and 10 (*P*  = 0.047). On day 10, all of the 15 test-positive samples in group AB related to *Te. circumcincta*, and three to *Trichostrongylus*, whereas *C. ovina* was not detected (see Table 1). In group MP, of the 20 samples that were test-positive by PCR on day 0, three were test-positive for *Te. circumcincta* only on day 10. In contrast, all 19 samples from group MPC were test-positive by PCR on day 0 and also on day 10 (*P*<0.001). The melting-curve analysis of all 301 amplicons produced in this study (irrespective of experimental group) as well as selective sequencing and comparison of resultant sequence tags (*n*  = 26) with reference sequences demonstrated unequivocally the specificity of both the amplicons and the PCR conditions employed.

### Comparison of Results Achieved by Routine Larval Culture (LC) and Total Worm Counts (TWC) with those from Molecular Testing

**Figure 1:**
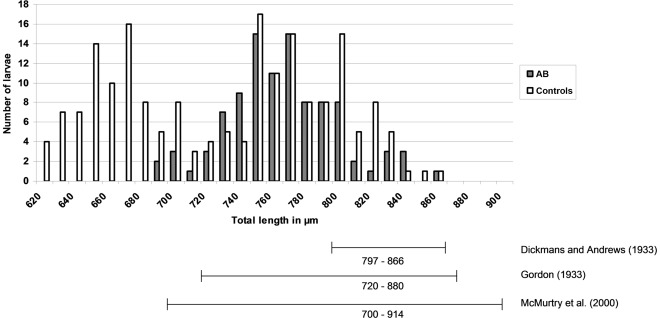
Histogram for the distribution of larval body-length. Distribution of the total lengths of third-stage larvae (L3) with a sheath extension of 30–40 µm, and the identification of *Teladorsagia circumcincta* L3s with respect to their total length (µm), as given by different authors [18]–[20].

On day 10, a pooled faecal sample representing all animals in each experimental group was tested by LC (Table 2). L3s of *Te. circumcincta* and *Trichostongylus* were identified in cultures representing three of the four experimental groups, and no larvae were detected for group MP. L3s of *Chabertia*/*Oesophagostomum* were identified in cultures representing both control groups (ABC and MPC) but not in the others. Morphometric comparisons of these L3s, according to Gordon [19] and McMurtry [20], consistently inferred *Te. circumcincta* as the most abundant parasite for each group, followed by *Trichostrongylus* spp., whereas *Chabertia*/*Oesophagostomum* were least abundant. This relationship was most pronounced in group AB, wherein >90% of the L3s were identified as *Te. circumcincta*. Measurements of total body length of L3s with a sheath extension of 30–40 µm inferred *Te. circumcincta* and *Trichostrongylus* spp. in groups ABC and MPC, and mainly *Te. circumcincta* in group AB (Fig. 1). These findings were similar to the results achieved by PCR testing of individual faecal samples from each of the four groups of sheep, although LC appeared to under-estimate the contribution of *Chabertia*/*Oesophagostomum* relative to the PCR, which can be explained by the ‘sensitivity’ of the molecular method. Notably, morphometric boundaries, as defined by Dikmans and Andrews [18], yielded results that were markedly different from those achieved using the criteria of Gordon [19] and McMurtry [20], with L3s of *Trichostrongylus* predicted as being most abundant in all cultures.

**Table 2:** Larval culture results.

		Reference (length in µm)				
		Dikmans and Andrews	Gordon	McMurtry		
Group^a^	Genus	(797–866)	(720–880)	(700–914)	xL3^c^	PCR (Ct)
ABC	*Teladorsagia*	20	56	65	54	17/20 (22.97)
	*Trichostrongylus*	78	42	33	40	17/20 (23.06)
	*Chabertia/Oesophagostomum*	2	2	2	6	13/20 (25.30)
AB	*Teladorsagia*	18	91	98	92	15/19 (24.61)
	*Trichostrongylus*	82	9	2	8	3/19 (25.53)
	*Chabertia/Oesophagostomum*	0	0	0	0	0/19 (N/A^b^)
MPC	*Teladorsagia*	16	48	54	52	15/20 (23.36)
	*Trichostrongylus*	76	44	38	40	19/20 (21.39)
	*Chabertia/Oesophagostomum*	8	8	8	8	15/20 (22.54)
MP	*Teladorsagia*	0	0	0	0	3/20 (26.18)
	*Trichostrongylus*	0	0	0	0	0/20 (N/A)
	*Chabertia/Oesophagostomum*	0	0	0	0	0/20 (N/A)

Larval culture results, following anthelmintic treatment (day 10), showing the percentage of different species (%), as determined by exsheathment and total body length measurement according to different authors [18]–[20]. Also shown are the numbers of species detected by PCR and mean cycle threshold value (Ct).

a Groups of sheep assigned as ABC (albendazole-untreated control), AB (albendazole treated), MPC (monepantel-untreated control), MP (monepantel treated).

b No data available.

c Exsheathed third-stage larvae.

To provide an independent comparison of LC and PCR, we conducted a routine TWC on 30 sheep (representing animals randomly-selected from each of the four groups). Because test-positive results in LC and PCR are dependent on the presence of eggs in faeces, TWCs related to the numbers of adult female worms in individual sheep, although worms of both sexes were counted (see Table 3). TWC data revealed the presence of females of *Te. circumcincta, Trichostrongylus* spp. (*T. axei, T. vitrinus, T. colubriformis*) and *C. ovina* in 21 (70%), 16 (53%) and eight (27%) of the sheep examined, respectively (Table 3). Moreover, in group AB, all sheep harboured *Te. circumcincta* (60–7340 females), whereas just three, low intensity infections of *Trichostrongylus* (50–160 females) were found, and no *Chabertia* or *Oesophagostomum*. These results are consistent with those achieved by PCR and LC using the morphometric criteria defined by Gordon [19] and McMurtry [20].

**Table 3:** Total worm count results.

			Abomasum				Small intestine				Large intestine			
Sheep no.	Group^a^	EPG	*Teladorsagia* females	all adults	L4	Ct	*Trichostrongylus* females	all adults	L4	Ct	*Chabertia* females	all adults	L4	Ct
1394	ABC	310	770	1960	10220	22.72	5000	8090	1200	20.72	1	1	0	27.16
1452	ABC	150	800	1200	8150	20.95	2460	3850	0	18.61	4	8	0	22.31
1338	ABC	40	200	520	1160	26.38	700	1140	0	25.34	6	7	0	24.48
1434	ABC	50	920	1620	2840	24.04	80*	100	0	40*	0	0	0	40
1353	ABC	30	300	450	11450	24.76	1460	2170	0	23.42	0	0	0	40
1330	ABC	530	850*	1300	5350	40*	6050*	9050	200	40*	3	3	0	26.82
1385	ABC	110	680	1120	1580	23.47	640	970	0	23.45	3	4	0	23.73
1429	ABC	280	3360	7200	30120	16.03	3420	4540	500	22.67	0	0	0	40
1403	ABC	120	1000	1950	7200	23.41	1506	2286	128	23.74	5	8	0	22.12
1427	AB	110	7340	11280	12500	21.91	60*	120	40	40*	0	0	0	40
1315	AB	50	1350	2200	5050	22.57	50*	100	0	40*	0	0	0	40
1390	AB	120	980	1660	600	22.4	0	20	0	40	0	0	0	40
1417	AB	60	60	80	1100	25.68	0	0	0	40	0	0	0	40
1305	AB	10	400	600	14500	25.1	160	200	440	25.51	0	0	0	40
1306	AB	40	816	1152	624	22.86	0	48	0	40	0	0	0	40
1409	AB	90	2600	4000	2250	24.5	0	0	0	40	0	0	0	40
1368	AB	30	80	160	160	24.6	0	20	0	40	0	0	0	40
1442	AB	30	200	300	5600	19.74	0*	0	0	27.03*	0	0	0	40
1313	MPC	750	1480	2640	820	18.53	10760	19880	1760	19.73	1	3	0	22.1
1345	MPC	1310	11200	17360	12460	21.92	1540	2460	0	23.66	7	11	0	22.18
1395	MPC	420	1800	3000	20850	20.94	11850	17750	1100	19.1	0	0	0	40
1387	MP	0	0	0	0	40	0	20	0	40	0	0	0	40
1415	MP	0	0*	0	0	26.14*	0	0	0	40	0	0	0	40
1443	MP	10	0*	0	0	25.5*	20*	20	0	40*	0	0	0	40
1379	MP	0	0	0	0	40	0	0	0	40	0	0	0	40
1362	MP	30	0	0	0	40	0	0	160	40	0	0	0	40
1326	MP	0	0	0	0	40	0	0	0	40	0	0	0	40
1451	MP	10	0	0	0	40	0	0	0	40	0	0	0	40
1399	MP	0	0	0	0	40	0	0	0	40	0	0	0	40
1445	MP	10	0	0	0	40	0	0	0	40	0	0	0	40

Total worm count results for 30 sheep following anthelmintic treatment. Corresponding faecal egg counts are given in eggs per gram (EPG) and PCR results, as cycle threshold values (Ct). Discrepancies between total worm count and PCR results indicated by asterisk. Negative PCR results are indicated by the number 40.

a Groups of sheep assigned as ABC (albendazole-untreated control), AB (albendazole-treated), MPC (monepantel-untreated control), MP (monepantel-treated).

Indeed, usually there was a close agreement between the routine TWC and PCR results achieved for each individual sheep examined using both methods. PCR analysis detected infections with *Te. circumcincta, Trichostrongylus* and *C. ovina* in 22 (73%), 12 (40%) and eight (27%) of the sheep examined, respectively (Table 3). Although *Te. circumcincta* was detected by PCR in three of 20 faecal samples from group MP on day 10, no adult worms of this species were detected by TWC in two of these three sheep (i.e., nos. 1415 and 1443) for which TWC reference data were recorded. Similarly, although *Trichostrongylus* DNA was detected by PCR in a sample from sheep no. 1442 from group AB, no worms were detected by TWC in the same sheep. Nonetheless, adult females of *Te. circumcincta* and *Trichostrongylus* (i.e. *T. axei* and *T. vitrinus*) were detected by TWC in sheep no. 1330 from group ABC, but DNAs from these parasites were not detected in the faeces from this sheep by PCR on day 10. *Trichostrongylus* DNA was detected by PCR in the faeces from 12 sheep with 160 to 11,850 female worms per sheep, but not in four sheep (nos. 1443, 1427, 1315 and 1443) in which <100 adult female worms per sheep were found. In spite of some differences in results between PCR and TWC, the diagnostic performance of the PCR was high. Using TWC as a reference method, PCR achieved an overall sensitivity ( =  the ability to detect a patent infection involving female worms of any of the species or genera being tested for) of 100%, a specificity of 87.5% and a Kappa value of 0.91 (Table 4). Kappa calculated for each PCR for each species or genus of parasite showed substantial to perfect agreement [26] with TWC results. During this study, the assay sensitivity could not be calculated for the species of *O. venulosum* and *H. contortus*, as these parasites were not detected in any of the sheep using any of the diagnostic methods employed.

**Table 4:** Determined assay performance.

Parasite species	(n)	Sensitivity	[95% CI]	(n)	Specificity	[95% CI]	Kappa	[95% CI]	Female worms
*Teladorsagia circumcincta*	(20/21)	95.2%	[76.2–99.9]	(7/9)	77.8%	[40.0–97.2]	0.75	[0.5–1.0]	≥60
*Trichostrongylus* spp.	(11/16)	68.8%^a^	[41.3–89.0]	(13/14)	92.9%	[66.1–99.8]	0.61	[03–0.9]	≥20
*Trichostrongylus* spp.	(11/12)	91.7%	[61.5–99.8]	(17/18)	94.4%	[72.7–99.9]	0.86	[0.7–1.0]	≥100
*Chabertia ovina*	(8/8)	100%	[63.1–100]	(22/22)	100%	[84.6–100]	1.00	(1.0–1.0)	≥1
*Oesophagostomum venulosum*	(0/0)	N/A^b^	N/A	(30/30)	100%	[88.4–100]	N/A	N/A	N/A
*Haemonchus contortus*	(0/0)	N/A	N/A	(30/30)	100%	[88.4–100]	N/A	N/A	N/A
Total	(22/22)	100%	[84.6–100]	(7/8)	87.5	[47.4–99.7]	0.91	[0.7–1.0]	N/A

PCR assay performance and direct comparison with total worm count results. Shown are the calculated diagnostic sensitivity and specificity of the PCR, the number of cases identified as positive or negative in comparison to total worm counts (n), calculated Kappa values and their 95% confidence interval (CI). Also shown are the minimum number of species females as detected by PCR and the total diagnostic performance (the ability to detect infections involving the presence of any females of the target species by PCR).

a all PCR false-negative test results related to TWC results of <100 female worms per sheep.

b No data available.

## Discussion

The diagnostic performance of the molecular assay assessed herein was high (sensitivity 100% and specificity 87.5%) in relation to TWC. Only for a small number of sheep there was a disagreement in the results between the two tests. Of the 136 faecal samples that contained strongylid eggs, only ten samples were test-negative by PCR (with no evidence of inhibition). Notably, for all PCR test-negative samples, EPG was <250, and all but one were collected from sheep following anthelmintic treatment. Microscopic examination revealed an abnormality in eggs and their shells from these samples, suggesting that the eggs were not viable and that DNA degradation led to these ‘false-negative’ PCR results. However, egg loss during flotation may also have contributed, suggesting that the direct isolation of DNA from faecal matter should be explored. Four sheep with ∼100 adult female worms of *Trichostrongylus* (*T. axei* and *T. vitrinus*) were PCR-test negative for this genus. Three of these four sheep were in groups AB and MP, suggesting that anthelmintic treatment led to a reduction in fecundity or affected the ability of the female worms to produce intact eggs, although this was not apparent upon morphological examination of these worms following TWC. Alternatively, the eggs themselves, and their DNA, may have undergone degradation in the gastrointestinal tract during or following treatment. Furthermore, the detection of low intensity infections by faecal flotation can be challenging due to the dilution and uneven distribution of eggs in the faeces of the host as well as daily variations in egg excretion [27], limiting the sensitivity of the faecal flotation approach (i.e., from a single 4 g sample collected at one time point). These latter statements are also supported by the technical limitation of FECRT, which only provides information on the effect of treatment on the reproduction of female worms rather than providing direct evidence of their effective removal [7]. It is most likely that the diagnostic sensitivity of individual assays is influenced by the reproductive potential of different species, so that eggs from highly fecund species (such as *H. contortus*, *O. venulosum* and *C. ovina*) can be more readily detected in sheep with small worm burdens than those for species with low fecundity (e.g., *Te. circumcincta* and *Trichostrongylus*) [28]. A similar restriction may apply also to the detection of parasite DNA by PCR, such that prepatent and very low-level infections might only be detectable by necropsy.

False-positive results were uncommon. In all nine cases, for which amplicons were produced from faecal samples in which no strongylid eggs were detected, subsequent sequencing confirmed, unequivocally, their specific identity, showing the limited sensitivity of the McMaster flotation method, also consistent with our previous field study [13]. In addition, in three samples found to be test-positive by PCR (and verified by direct DNA sequencing), we detected no evidence of infection based on routine total worm count (TWC). Given that TWC is based on sampling and examining sub-aliquots of gut contents, we infer this discrepancy to relate to limitations in the sensitivity of TWC rather than an issue with the PCR assay. Indeed, because only one 20-th of the total gut wash (1 litre) is examined for worms, the theoretical minimum number of worms detectable using this approach is 20 (which equates to the observation of 1 worm in the aliquot examined). Moreover, although it has been shown [29] that the vast majority of trichostronglyids infecting the small intestine of sheep are usually present in the first six meters, the effect of sub-sampling from the gut wash likely compromises the accuracy of the method. This aspect has not been critically assessed to date and, thus, warrants detailed investigation. The inaccuracy related to the probability of detecting worms in a sub-sample would be particularly pronounced in sheep with low numbers of worms, potentially leading to significant over- or under-estimation of infection intensity. Acknowledging these issues, we selected TWC as the reference method, because it is recognized as the ‘gold-standard’ for the diagnosis of infections with gastrointestinal helminths [30].

Consistent with EPG and TWC data, the present PCR results did not show any evidence of resistance to monepantel in gastrointestinal strongylids, supporting previous reports of the efficacy of this new anthelmintic [31], [32]. In contrast, the PCR assay did provide evidence of a reduced susceptibility to benzimidazoles (i.e., albendazole) in one or more nematode species in the population of sheep in this study. These results were expected, based on the history available for this farm (J. Larsen, unpublished) and also the relatively high prevalence of benzimidazole resistance in sheep in Australia [33], [34], and were supported by coproscopic and TWC data. Indeed, following albendazole treatment, 17 of 19 sheep were shown to harbour strongylids based on McMaster flotation (day 10) and, despite a notable overall reduction in EPG, the number of samples with eggs increased (between days 0 and 10) from 15 to 17 in group AB. PCR-based testing detected infection/s in 15 of the 19 sheep on day 10, with subsequent sequencing indicating the presence of *Te. circumcincta* in all and *T. colubriformis* in three sheep with EPGs of 0–120 following treatment. Although *C. ovina* was initially detected in eight sheep in group AB (on day 0), this nematode was not detected following albendazole treatment (on day 10), providing no evidence of drug resistance in this species. Based on these data, a specific diagnosis of resistance to albendazole was possible and indicated a predominant link to *Te. circumcincta* and, to lesser extent, *T. colubriformis*.

Using the criteria defined by Gordon [19] and McMurtry [20], the LC results, following anthelmintic treatment, were similar to those achieved by PCR. Nonetheless, the morphological identification of L3 stages (based on total length and caudal morphology) is complicated by the similarity between *Teladorsagia* and *Trichostrongylus* species. Most notably, when using the criteria defined by Dikmans and Andrews [18], the majority of the L3s from LC for group AB were identified as *Trichostrongylus* rather than *Te. circumcincta*, thus leading to an entirely different diagnosis and conclusion regarding resistance. The inference that *Trichostrongylus* had the highest level of resistance to albendazole was neither supported by the PCR-test results nor the TWC data, emphasizing the problems associated with the use of LC. The limitations in the differentiation of some parasites following LC are reinforced by findings that host (e.g., immune response) and/or environmental factors (e.g., climate and/or the availability of appropriate nutrients for first- and second-stage larvae) can influence the length of the developing L3s [20], obviously, leading to further challenges for a correct diagnosis of resistance in the context of FECRT combined with LC. Because PCR relies on the use of species/genus-specific DNA markers, such factors do not adversely impact on its application and reliability. Furthermore, although PCR can detect *C. ovina* and *Oesophagostomum* and differentiate them, L3s of these parasites cannot be delineated morphologically [17]. In addition to these technical considerations, LC has significant practical limitations compared with a PCR-based method, particularly in relation to time-efficiency and the cost of testing.

In conclusion, based on the results of the present and previous studies [13], [23], we have consistently demonstrated that our PCR approach, employing genetic markers in nuclear rDNA, is specific for strongylid nematodes [12], [25] and achieves the sensitivity required for efficient diagnosis of naturally acquired strongylid infections in sheep. In addition, the present investigation provides strong evidence that this molecular assay can support FECRT for the detection of anthelmintic resistance in strongylid populations, thus eliminating the need for LC. A molecular assay that directly detects drug resistance, and, thus, replaces or at least reduces the need for FEC-based reduction trials altogether, would be a major, additional step forward. However, current tests are limited to the detection of benzimidazole resistance based mainly on three main mutations (linked to amino acid positions 167, 198 and 200) in the beta tubulin gene [7], [35], but neither levamisole nor macrocyclic lactone resistance, which appear to be multi-faceted and polygenic [36]. Therefore, molecular assays for the direct detection of drug resistance will likely be limited until the genetics and genomics of resistance are much better understood. In contrast, coupled to current FECRT methods, our specific PCR assay provides a rapid, efficient and universally applicable tool for the diagnosis of resistance and the early detection of residual populations of worms in sheep following treatment, possibly reflecting an early emergence of resistance.

Taken together, our results show that the present PCR is useful as a rapid approach for routine *intra vitam* diagnosis of strongylid infections in sheep and, combined with conventional FECRT, for assessing the emergence of anthelmintic resistance, without the need for additional costly and time-consuming *ante mortem* (i.e., LC) or *post mortem* (TWC) analyses. Further applications of PCR might include its use for assessing the monospecificity of cultures used for a range of experimental investigations of strongylids and mechanisms of drug resistance in particular species or, for instance, to assess the status of parasitism in flocks of sheep destined for import/export. Given the broad applicability of such a molecular-diagnostic assay, our current focus is now on adapting it to a semi-automated platform for routine application in a service laboratory setting.
